# Cariprazine as a candidate for drug repurposing in anorexia nervosa: a mechanistic hypothesis grounded in neuroimaging evidence

**DOI:** 10.3389/fphar.2026.1877615

**Published:** 2026-07-14

**Authors:** Attila Kovacs, Krisztina Csapo, Gabor Andrassy, Csaba Papp, Rudolf Gesztelyi, Judit Zsuga

**Affiliations:** 1 Department of Psychiatry, Faculty of Medicine, University of Debrecen, Debrecen, Hungary; 2 Institute for Primary Care and Health Promotion of Debrecen, Clinical Center, University of Debrecen, Debrecen, Hungary; 3 Department of Pharmacology and Pharmacotherapy, Faculty of Medicine, University of Debrecen, Debrecen, Hungary; 4 Centre for Translational Neuropsychiatry, Clinical Center, University of Debrecen, Debrecen, Hungary

**Keywords:** anhedonia, anorexia nervosa, cariprazine, dopamine D3 receptor, drug repurposing, neuroimaging, reward, serotonin

## Abstract

Anorexia nervosa (AN) carries the highest mortality of any psychiatric disorder, yet no pharmacological agent has received regulatory approval for its treatment in adults. Multimodal neuroimaging has now revealed a coherent neurobiological architecture: functional MRI documents blunted ventral striatal and amygdalar responses to food, alongside excessive prefrontal–cingulate engagement; PET corroborates trait-level reductions in 5-HT2A binding, elevations in 5-HT1A binding across cingulate, frontal, parietal, temporal, and dorsal raphe regions, and compensatory upregulation of D2/D3 receptors in the anteroventral striatum; SPECT identifies persistent temporal and cingulate hypoperfusion; and EEG reveals attenuated P300 amplitudes, delayed N2 latencies, and parieto-occipital theta hyperarousal. We propose that cariprazine—a dopamine D3-preferring D3/D2 receptor partial agonist with 5-HT1A partial agonism and 5-HT2A/2B antagonism—confers a uniquely matched pharmacological toolkit for this disorder. Cariprazine’s D3 partial agonism is predicted to restore mesolimbic dopamine signaling, thereby reinstating incentive salience to food cues; its 5-HT1A stabilizer property attenuates the trait-amplified corticolimbic inhibitory tone underlying harm avoidance and anxiety; its prefrontal glutamate-modulating action attenuates excessive cognitive control over eating, and chronic administration confers substrate-dependent set-point recalibration of the upregulated mesolimbic D3 receptor pool. Furthermore, cariprazine’s favorable metabolic profile (number needed to harm for clinically significant weight gain of 34 *versus* 6 for olanzapine) renders its safety burden meaningfully lower than that of currently used agents. In summary, the receptor pharmacology of cariprazine maps with notable specificity onto the neuroimaging-defined substrate of AN, providing a mechanistically grounded rationale for prospective clinical investigation of cariprazine as a candidate pharmacotherapy for this otherwise intractable disorder.

## 1 Introduction

### 1.1 Background

Anorexia nervosa (AN) is characterized by persistent restriction of energy intake, intense fear of weight gain, and a disturbed perception of body weight or shape (DSM-5). It carries a standardized mortality ratio of approximately 5.86, making it one of the most lethal psychiatric illnesses, with an estimated 5%–10% of patients dying from medical complications or suicide within 10 years of diagnosis and the cause of death being suicide in 1 case out of 5 cases (Arcelus et al., 2011; Franko et al., 2013). The disorder assumes a lifetime prevalence of approximately 0.1%–3.6% and 0%–0.3% among women and men, respectively (Van Eeden et al., 2021). Despite this severity, no pharmacological agent has received regulatory approval for the treatment of AN in adults (Crow, 2019). The most rigorously studied agent, olanzapine, produced a statistically significant but clinically modest increase in BMI of 0.6 kg/m^2^ at 16 weeks, with no significant benefit on eating disorder psychopathology—at the cost of substantial metabolic and weight-gain liabilities that further complicate its use (Attia et al., 2019). Antidepressant trials have consistently failed to demonstrate efficacy in underweight AN patients (Crow, 2019), and beneficial effects are limited to weight-restored AN patients. Thus, there is, accordingly, an urgent and unmet clinical need for mechanistically grounded pharmacotherapy.

Over the past 2 decades, neuroimaging research has transformed our understanding of AN from a primarily psychosocial disorder to that with reproducible neurobiological substrates. Functional MRI studies document blunted reward circuitry responses to food; PET studies reveal persistent serotonergic (5-HT) and dopaminergic (DA) receptor abnormalities even after weight restoration; SPECT studies consistently identify hypoperfusion in temporal, cingulate, and frontal regions, and EEG studies show cortical arousal and deficits in inhibitory control (see below). Collectively, these findings implicate the mesolimbic dopamine system, the prefrontal–cingulate network, the insular cortex, and the brainstem serotonergic nuclei as the principal substrates of the disorder’s core symptoms.

Alteration of the dopaminergic system has been articulated in recovered AN patients as trait-level elevations in D2/D3 binding have been documented in recovered patients (Frank et al., 2005) as evidence of chronic hypodopaminergia in the mesolimbic system, while an addiction-like behavioral phenotype of active AN has also been described (Kaye et al., 2009). Evidence at both preclinical and clinical levels corroborates the view that mesolimbic dopaminergic responsiveness in AN is cue-selective rather than uniformly suppressed: in the activity-based anorexia rodent model, accumbal dopamine release was not elevated during the initiation of food-anticipatory behavior, despite marked starvation and hyperactivity (Verhagen et al., 2009), whereas chemogenetic activation of the ventral tegmental area-to-nucleus accumbens dopaminergic projection rescued body weight by selectively augmenting food intake (Foldi et al., 2017). Conversely, fMRI demonstrates exaggerated ventral striatal activation in response to underweight body-image stimuli even in early-illness adolescent AN (Fladung et al., 2013). It seems reasonable to suggest that mesolimbic hypodopaminergia in AN is selective to food cues, while non-food cues—particularly weight-loss-related visual feedback and exercise—recruit a preserved or augmented dopaminergic response, generating the addiction-like reinforcement loop described by Ziemichód et al. (2026). This dual-cue formulation reconciles the seemingly divergent findings of trait-level hypodpaminergia and addiction-like behavior.

Serotonergic system perturbations documented in AN are not a mere epiphenomon but rather constitute an integrated trait-level receptor-imbalance architecture, with 5-HT1A receptor predominance over 5-HT2A receptors. The serotonergic signature of AN is organized along three mechanistically distinct axes: an axis of ligand availability [CSF 5-hydroxi-indo-acetic-acid (5-HIAA) and tryptophan metabolism], an axis of receptor density (5-HT1A and 5-HT2A), and an axis of network-level modulation (cortical excitability and raphe–corticolimbic connectivity). This concept explains the paradox of AN—food restriction is experienced as ego-syntonic and affectively relieving rather than aversive.

An increase in serotonin levels was proposed previously based on the fact that cerebrospinal fluid concentration of the 5-HT metabolite 5-HIAA was shown to be elevated in long-term weight-restored AN patients relative to controls (Kaye et al., 1991). The hyperserotonergic phenotype is corroborated by the now-classical demonstration that acute tryptophan depletion—which lowers central 5-HT synthesis—produces a paradoxical anxiolytic, rather than dysphoric, response in ill AN patients (Kaye et al., 2003). Furthermore, it may be postulated that patients with AN premorbidly inhabit a state of aversive serotonergic overactivity and that caloric restriction—by lowering peripheral tryptophan availability—functions as a self-administered pharmacological strategy for dampening this aversive tone (Kaye et al., 2009).

Furthermore, a shifted balance toward 5-HT1A predominance over 5-HT2A has also been described in the context of AN (Kaye et al., 2009). As 5-HT1A and 5-HT2A receptors exert opposing effects on cortical pyramidal neuron excitability (Araneda and Andrade, 1991), with the former hyperpolarizing *via* potassium conductance and the latter depolarizing *via* Gq-coupled signaling, a trait-level shift toward 5-HT1A predominance, therefore, renders corticolimbic pyramidal neurons biased toward inhibitory-hyperpolarizing gating of affective input, producing the anxious, harm-avoidant, threat-biased cognitive-affective phenotype. Finally, the network-modulation offered by the elevated 5-HT1A binding at somatodendric sites on the dorsal raphe itself carries several implications for the AN symptom domains as activation of these autoreceptors produces a tonic negative-feedback restraint upon ascending serotonergic firing and thus modulates the global delivery of 5-HT to forebrain targets (see below).

Cariprazine (Vraylar/Reagila) is a dopamine D3-preferring D3/D2 receptor partial agonist, also acting as a partial agonist at the serotonin 5-HT1A receptor and an antagonist at 5-HT2A and 5-HT2B receptors (Citrome, 2016). It is approved for schizophrenia and acute manic, mixed, and depressive episodes of bipolar I disorder in several countries and has demonstrated efficacy against negative symptoms, anhedonia, and cognitive deficits (Koziej et al., 2024; Citrome, 2018). Its unique receptor profile—particularly its approximately 10-fold preferential affinity for D3 over D2 receptors (Kiss et al., 2010)—maps with notable specificity onto the neurobiological targets identified by neuroimaging in AN.

In the following sections, we compile evidence to support the hypothesis that cariprazine’s receptor pharmacology is uniquely suited to the neurobiological substrate of AN, by systematically reviewing the multimodal neuroimaging abnormalities of the disorder and mapping each finding onto the mechanistic predictions arising from cariprazine’s pharmacodynamic profile. To establish the relevance of the neuroimaging findings with respect to clinical symptoms in the domains of AN, neuroimaging findings are reviewed across four modalities—functional MRI, EEG, PET, and SPECT—each of which adds to our understanding regarding specific neural circuit perturbations. To render these findings interpretable and to underscore their full clinical significance, first, the relationship between AN’s core clinical symptom domains and their neurobiological substrates are described. AN is not a unitary syndrome but a cluster of five clinically and neurobiologically distinct symptom domains—food reward hyposensitivity, harm avoidance and anxiety, body image distortion, cognitive rigidity, and impaired interoception—each of which plays a significant role in perpetuating the disorder and is now understood to reflect discrete changes in identifiable neural circuits (Kaye et al., 2013; Treasure et al., 2015).

### 1.2 Food reward hyposensitivity and motivated restriction

The cardinal clinical feature of AN—the deliberate and persistent restriction of caloric intake in the context of progressive emaciation—cannot be adequately explained by a primary loss of appetite. Rather, neurobiological evidence increasingly posits that tonic hypodopaminergia in the mesolimbic system renders food stimuli motivationally inert, stripping it of their incentive salience in Berridge’s framework of dopaminergic “wanting” (Kaye et al., 2013; Berridge and Robinson, 2016). This is corroborated by meta-analytic evidence that anhedonia is significantly more pronounced in AN than in healthy controls (Dolan et al., 2022). Additionally, clinical observations indicate that dietary restriction is frequently experienced as ego-syntonic—even affectively relieving—in AN (Ziemichód et al., 2026). Patients report reduced anxiety and dysphoria following food avoidance, a pattern consistent with relief from the aversive consequences of abnormal serotonergic tone rather than the experience of hedonic reward from eating (Kaye et al., 2009); conversely, the addiction-like reinforcement of restriction itself—sustained by the visible effects of weight loss and by compulsive physical activity—has been articulated as a parallel dopaminergic mechanism (Ziemichód et al., 2026). Conversely, fMRI demonstrated exaggerated ventral striatal activation in response to underweight body-image stimuli even in early-illness adolescent AN (Fladung et al., 2013). Thus, it is suggested that mesolimbic hypodopaminergia in AN is selective to food cues, but non-food cues—particularly weight-loss-related visual feedback and exercise—are able to show a preserved or augmented dopaminergic response, generating the addiction-like reinforcement loop described by Ziemichód et al. (2026).

Food reward hyposensitivity may be indirectly linked to elevated serotonin levels as the anxiolytic reinforcement conferred by tryptophan-limiting starvation renders food restriction behaviorally rewarding, thereby making restriction not a failure of appetitive regulation but a rationally deployed behavioral anxiolytic (Kaye et al., 2003). Accordingly, this symptom domain maps directly onto the fMRI finding of blunted ventral striatal and amygdala activation to food reward and the PET finding of elevated D2/D3 receptor binding potential in the anteroventral striatum—both reviewed in Sections 2.1 and 2.3, respectively—and it renders the mesolimbic dopamine system the central pharmacological target of the present hypothesis.

### 1.3 Harm avoidance, anxiety, and perfectionism

Individuals with AN characteristically exhibit elevated harm avoidance, perfectionism, and chronic anxiety—traits that typically predate illness onset, persist after weight restoration, and have been documented in unaffected first-degree relatives, underscoring their status as neurobiological trait vulnerabilities rather than consequences of malnutrition (Treasure et al., 2015; Kaye et al., 2013). Clinically, harm avoidance manifests as an intense and irrational fear of weight gain, rigid dietary rules, and a marked intolerance of uncertainty and novelty (Frank et al., 2012). This domain has been associated with increased central serotonergic activity.

The receptor level changes are now substantially defined. PET evidence demonstrates increased 5-HT1A receptor binding in the cingulate, lateral, and mesial temporal cortex, the prefrontal cortex, the parietal cortex, and the dorsal raphe in ill AN patients (Bailer et al., 2007), alongside reduced 5-HT2A receptor binding in the mesiotemporal and cingulate cortex that persists after weight restoration (Frank et al., 2002; Bailer et al., 2004). These regional changes predict a specific functional consequence: potentiated 5-HT1A-mediated inhibitory tone dominates over attenuated 5-HT2A-mediated excitatory tone, resulting in net 5-HT1A-mediated inhibitory-hyperpolarizing gating of affective input and producing the anxious, harm-avoidant, threat-biased cognitive–affective phenotype central to AN (Kaye et al., 2009). Furthermore, the network modulation associated with elevated 5-HT1A binding at somatodendric sites within the dorsal raphe itself maps predominantly onto the harm avoidance and anxiety symptom domain (Bailer et al., 2007).

The SPECT finding of anterior cingulate hypoperfusion, reviewed in Section 2.4, is in line with impaired error monitoring and heightened harm detection—the neural substrate of the relentless self-corrective drive that characterizes AN. The serotonergic correlate of harm avoidance, however, presents a two-tiered signature that warrants explicit articulation. At the between-group level, [18F]altanserin binding is reduced in recovered AN relative to controls (Frank et al., 2002; Bailer et al., 2004), corroborating the trait-level downregulation of 5-HT2A receptor density. At the within-group level, residual 5-HT2A binding was positively correlated with harm avoidance scores in the cingulate and temporal regions in recovered (bulimic type) patients with AN (Bailer et al., 2004) and in the suprapragenual cingulate, frontal, and parietal regions of patients with ill AN (Bailer et al., 2007). Although these two findings are superficially in tension, they jointly provide a coherent picture: in AN, the substrate exhibits a global compression of the 5-HT2A receptor pool, and within this compressed pool, residual receptor density tracks the severity of the harm-avoidant phenotype. Accordingly, the serotonergic substrate of harm avoidance in AN is not a uniform deficit signal but a population-distributed feature in which both the absolute reduction and the gradient across the residual pool may assume a clinical role.

### 1.4 Body image distortion

Among the most clinically salient and treatment-resistant features of AN is the perceptual and evaluative distortion of body image: patients experience themselves as overweight despite objective emaciation and show marked preoccupation with body shape and weight that assumes a self-referential, ruminative character (Treasure et al., 2015). This domain is important as it is highly resistant to psychotherapeutic intervention and predictive of relapse. It maps onto the SPECT findings of the right superior parietal cortex and temporoparietal junction activation during body image exposure tasks (Beato-Fernández et al., 2009)—implicating these regions in the active maintenance of a distorted body schema—and onto the fMRI findings of increased right parahippocampal activation during self-referential processing in AN patients (Su et al., 2021), corroborating the notion that contextual body-related memory is disproportionately engaged, possibly rendering the distorted self-representation highly resistant to experiential updating.

### 1.5 Cognitive rigidity and impaired set-shifting

AN is characterized by an unusually rigid, rule-governed approach to eating and behavior more broadly: patients exhibit marked cognitive inflexibility, difficulty in shifting cognitive set, and an abnormal tendency toward detail-focused, rather than global, information processing (Kaye et al., 2013). These traits play a clinical role in the maintenance of restrictive eating patterns by rendering the patient unable to revise entrenched dietary rules in response to corrective feedback (Ziemichód et al., 2026). Cognitive rigidity maps onto the increase in prefrontal 5-HT1A receptor binding and reduction in 5-HT2A receptor binding since 5-HT2A signaling in prefrontal pyramidal neurons subserves cognitive flexibility and set-shifting, while 5-HT1A-mediated pyramidal hyperpolarization biases the prefrontal network toward stable, perseverative activity patterns rather than flexible task-switching (Kaye et al., 2009). Moreover, the network modulation offered by the elevated 5-HT1A binding in the dorsal raphe itself is a potential factor regarding cognitive rigidity through the impaired efficiency of attentional gating under conditions of chronic cortical hyperarousal (Bailer et al., 2007). Neurobiologically, this symptom domain maps onto the fMRI finding of excessive prefrontal and dorsal striatal engagement during reward and habit tasks, reviewed in Section 2.1, and onto the EEG finding of attenuated P300 and delayed N2 components during inhibitory control paradigms, reviewed in Section 2.2. Together, these findings corroborate that cognitive rigidity in AN is not merely a psychological style but reflects measurable abnormalities in the efficiency of prefrontal inhibitory and attentional gating circuits (Yue et al., 2020; Berchio et al., 2022). Furthermore, the elevated theta activity in parieto-occipital regions identified by EEG, reviewed in Section 2.2, is in line with a state of chronic anxious hyperarousal that additionally hinders flexible attentional allocation.

### 1.6 Impaired interoception

A clinically underrecognized but neurobiologically central feature of AN is the failure to accurately detect and act upon internal bodily signals, including hunger, satiety, and visceral distress. Patients frequently report diminished awareness of hunger despite frank caloric deprivation, an inability to discriminate hunger from anxiety, and impaired recognition of satiety signals; these interoceptive deficits directly contribute to the perpetuation of restriction behaviors (Kaye et al., 2013). The 5-HT1A-mediated modulation of insular and hippocampal circuits that subserve interoceptive prediction contributes to impaired interoception (Kaye et al., 2009), and the network-modulation associated with elevated 5-HT1A binding in the dorsal raphe itself also contributes to impaired interoception through serotonergic modulation of insular interoceptive prediction circuits. The impaired interoception domain is accounted for by indirect 5-HT1A modulation of insular and raphe–insular circuits, whose trait-level high-gain signature distorts visceral prediction such that anxiety-related visceral noise supplants accurate hunger detection (Bailer et al., 2007; Kaye et al., 2009). This maps onto the fMRI finding of reduced insula activation during food stimuli (Bronleigh et al., 2022) and increased dorsal mid-insula activation during anxious rumination in weight-restored AN, with stomach-interoception activity correlating with eating-disorder psychopathology severity (Kerr et al., 2016), a finding consistent with a distorted, rather than absent, interoceptive signal, in which anxiety-related visceral noise overshadows accurate hunger detection.

In summary, AN’s five core clinical symptom domains—food reward hyposensitivity, harm avoidance and anxiety, body image distortion, cognitive rigidity, and impaired interoception—each find their neurobiological expression in reproducible perturbations of discrete neural circuits, as identified by multimodal neuroimaging. This mapping posits a compelling neurobiological architecture for an illness that has been conceptualized exclusively in psychosocial terms, and it establishes the framework within which the neuroimaging findings reviewed in the following sections attain their full clinical significance. The convergence of multiple independent modalities on a coherent picture of mesolimbic hypodopaminergia, serotonergic trait vulnerability, prefrontal overengagement, and limbic hypoperfusion shows a picture that, as argued in Sections 3 and 4, corresponds with notable specificity onto the pharmacodynamic profile of cariprazine.

## 2 Neuroimaging abnormalities in anorexia nervosa

**FIGURE 1:**
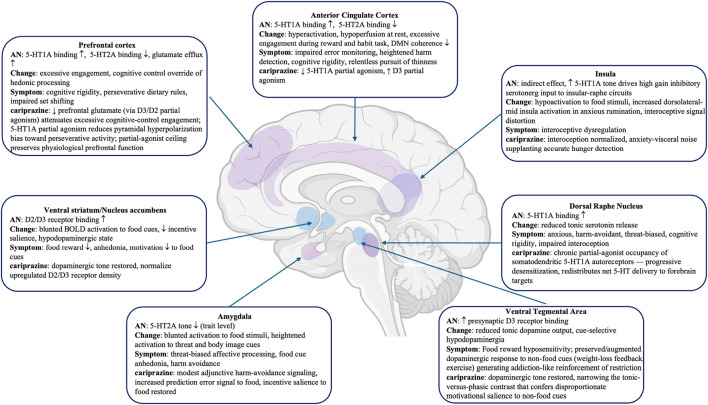
The cross-modality summary of neuroimaging findings and the predicted effects of cariprazine in AN. Abbreviations: 5-HT1A, serotonin receptor subtype 1A; 5-HT2A, serotonin receptor subtype 2A; AN, anorexia nervosa; BOLD, blood-oxygen-level-dependent signal; D2/D3, DMN, default mode network, dopamine receptor subtypes 2 and 3.

Multimodal neuroimaging provides a uniquely powerful means of mapping the neurobiological substrate of anorexia nervosa since each technique interrogates a complementary level of cerebral organization—functional activation, oscillatory dynamics, receptor-level pharmacology, and regional perfusion. Accordingly, the present section synthesizes evidence from four imaging modalities (fMRI, EEG, PET, and SPECT), each of which corroborates a distinct facet of the disorder’s neural architecture. Functional MRI documents perturbations of the mesolimbic reward circuit, prefrontal cognitive control, and insular interoception; EEG reveals deficits in inhibitory action monitoring, alongside trait-level cortical hyperarousal; PET identifies trait-stable serotonergic and dopaminergic receptor abnormalities that persist after weight restoration, and SPECT documents regional hypoperfusion in temporal, cingulate, and parietal cortices. It should be noted that the convergence of these independent modalities upon a coherent neurobiological signature underscores AN’s status as a disorder of identifiable circuit pathology rather than a primarily psychosocial phenomenon, thereby establishing the empirical substrate on which the cariprazine-based mechanistic hypothesis advanced in subsequent sections rests. The cross-modality summary of neuroimaging findings in AN are summarized in Figure 1 and Table 1.

**TABLE 1:** Cross-modality summary of neuroimaging findings in anorexia nervosa and their clinical correlates core clinical symptom domains of AN are as articulated by Kaye et al. (2013) and Treasure et al. (2015). Cells marked “—” denote no specific finding.

Clinical domain	fMRI	EEG	PET	SPECT
Food reward hyposensitivity	Blunted ventral striatal and amygdala activation during visual food processing; reduced insula activation (Bronleigh et al., 2022). Cue-selective rather than uniformly suppressed mesolimbic dopaminergic responsiveness, with exaggerated ventral striatal activation to underweight body-image stimuli (Fladung et al., 2013).	—	Elevated D2/D3 receptor binding potential in the anteroventral striatum, interpreted as compensatory upregulation in response to tonic mesolimbic dopamine hypofunction (Frank et al., 2005).	—
Harm avoidance and anxiety	—	Elevated parieto-occipital and temporal theta amplitude with reduced-alpha/increased-beta signature, interpreted as a state of chronic anxious cortical hyperarousal (Spalatro et al., 2021; Hatch et al., 2011; Heinbockel et al., 2021).	Increased 5-HT1A binding in cingulate, lateral and mesial temporal, prefrontal, parietal cortices, as well as in dorsal raphe; reduced 5-HT2A binding in the mesiotemporal and cingulate cortex, with binding potential positively correlated with harm avoidance (Frank et al., 2002; Bailer et al., 2004; Bailer et al., 2007).	Anterior cingulate hypoperfusion at rest in restricting-type AN, in line with impaired error monitoring and heightened harm detection (Naruo et al., 2001).
Body image distortion	Increased right parahippocampal activation during self-referential processing, reflecting disproportionate engagement of contextual body-related memory (Su et al., 2021).	—	—	Hyperactivation of the left parietal and right superior frontal cortex during filmed body image exposure, with the left parietal rCBF changes specifically associated with body distortion severity (Beato-Fernández et al., 2009).
Cognitive rigidity and impaired set-shifting	Excessive prefrontal and dorsal striatal engagement during reward and habit tasks; heightened dorsolateral prefrontal and anterior cingulate activation reflecting cognitive control override of hedonic processing (Bronleigh et al., 2022; Ehrlich et al., 2015).	Attenuated P300 amplitudes and delayed N2 latencies during inhibitory control paradigms; positive correlation of N200 latency and P300 amplitude with BMI (Yue et al., 2020; Berchio et al., 2022).	Prefrontal 5-HT1A elevation and 5-HT2A reduction biasing the prefrontal network toward stable, perseverative activity rather than flexible task-switching (Bailer et al., 2007).	—
Impaired interoception	Reduced insula activation in response to food stimuli (Bronleigh et al., 2022); increased dorsal mid-insula activation during anxious rumination, with stomach-interoception activity correlated with eating-disorder psychopathology severity in weight-restored AN patients (Kerr et al., 2016).	—	Indirect 5-HT1A modulation of insular and raphe–insular circuits, with trait-level high-gain signature distorting visceral prediction (Bailer et al., 2007; Bischoff-Grethe et al., 2013).	—

### 2.1 Functional MRI: reward, cognitive control, and interoception

The most replicated fMRI finding in AN is blunted neural activation in response to food stimuli. A coordinate-based ALE meta-analysis of nine fMRI studies on patients with anorexia nervosa vs. healthy controls (132 AN patients and 161 healthy control participants) demonstrated hypoactivation of the amygdala and the striatum—key nodes of the reward circuit—and of the insula during visual food processing in AN patients relative to the healthy controls (Bronleigh et al., 2022). These findings are consistent across studies and are in line with clinical observations of food-related anhedonia and the reported loss of hedonic values associated with eating (Dolan et al., 2022). Interpretation of blunted ventral striatal BOLD as a signal for altered dopaminergic function is inferential but nevertheless corroborated by the PET literature reviewed below. Accordingly, the mesolimbic dopamine system emerges as the principal neurobiological correlate of this reward hyposensitivity. Alongside this reward hyposensitivity, AN is paradoxically characterized by excessive prefrontal and dorsal striatal engagement: patients with acute and recovered AN show heightened activation in the dorsolateral prefrontal cortex and the anterior cingulate cortex during reward and habit tasks, a finding reflective of an unusually strong cognitive-control override of hedonic processing (Ehrlich et al., 2015). Interoceptive dysfunction is documented by increased dorsal mid-insula activation during anxious rumination in weight-restored restricting-type AN, with stomach-interoception activity correlating with eating-disorder psychopathology severity (Kerr et al., 2016). At the network level, a multimodal meta-analysis of 660 patients with AN and 740 controls found consistent reductions in resting-state functional activity in the bilateral anterior and median cingulate cortex and increased activity in the right parahippocampal gyrus (Su et al., 2021), with altered temporal coherence between the default mode network, precuneus, and dorsolateral prefrontal cortex consistent with the pervasive body-related rumination that characterizes the disorder (Cowdrey et al., 2014).

### 2.2 EEG: inhibitory control and cortical arousal

EEG event-related potential (ERP) studies on AN converge on deficits in inhibitory control indexed (Ramautar et al., 2004) by the N2 and P300 components. A study on 27 unmedicated AN patients using stop-signal tasks found attenuated P300 amplitudes and delayed N2 latencies across inhibitory demand conditions, with N200 latency and P300 amplitude positively correlated with BMI and N200 amplitude correlated with eating-disorder symptom severity (Yue et al., 2020). A systematic review of EEG in eating disorders confirmed reduced action-monitoring control—reflected by preparatory waves, N200, and P300—alongside fundamental alterations in posterior theta oscillations (Berchio et al., 2022). Resting-state oscillatory findings in AN include elevated slow-wave theta activity in parieto-occipital and temporal regions interpreted as a marker of cortical hyperarousal, alongside reduced-alpha and increased-beta power at rest (Spalatro et al., 2021); the posterior theta elevation persists after refeeding and thus behaves as a trait rather than a state marker (Hatch et al., 2011).

Furthermore, the network-level functional consequence of the elevated 5-HT1A binding documented at both postsynaptic corticolimbic and presynaptic dorsal raphe sites (Bailer et al., 2007) warrants explicit articulation. Activation of somatodendritic 5-HT1A autoreceptors produces a tonic negative-feedback restraint upon ascending serotonergic firing and thus modulates the global delivery of 5-HT to forebrain targets. The trait-amplified autoreceptor brake—conjoined with the cortical postsynaptic 5-HT1A elevation—produces a specific functional signature: any given amount of endogenous 5-HT generates a disproportionately large inhibitory cortical signal, while the presynaptic restraint on 5-HT output is also disproportionately strong. This dual amplification at presynaptic and postsynaptic loci yields an unusually noise-sensitive, high-gain inhibitory serotonergic system in which small perturbations of tryptophan availability or endogenous 5-HT release produce exaggerated swings in inhibitory cortical tone. The electrophysiological correlate of this network signature is the cortical hyperarousal documented in resting-state EEG, namely the elevated parieto-occipital and temporal theta amplitude with a reduced-alpha/increased-beta profile. It is plausible that the AN cortex, rendered unusually sensitive to serotonergic input by the receptor-density shift, manifests this sensitivity as a state of chronic anxious hyperarousal detectable at the population-oscillatory level.

### 2.3 PET: serotonin and dopamine receptor binding

PET neuroimaging has identified two principal trait-level receptor abnormalities in AN—serotonergic and dopaminergic—both obtained in weight-recovered patients to minimize confounds from starvation, thus reflecting enduring neurobiological vulnerabilities rather than consequences of malnutrition.

The most replicated PET finding is reduced 5-HT2A receptor binding in mesiotemporal and cingulate cortical regions. Using [18F]altanserin, Frank et al. (2002) demonstrated significantly reduced 5-HT2A binding in the amygdala, hippocampus, and cingulate cortex in 16 women recovered from restricting-type AN compared to 23 healthy controls. Parallel findings were reported by Bailer et al. (2004) in 10 women recovered from bulimia-type AN, who showed reduced [18F]altanserin binding potential in the left subgenual cingulate, left parietal cortex, and right occipital cortex, with binding potential positively correlated with harm avoidance and negatively correlated with novelty seeking. Bailer et al. (2007) further demonstrated, using [^11^C]WAY-100635, that ill AN patients exhibit increased 5-HT1A receptor binding in cingulate, lateral and mesial temporal cortex, prefrontal cortex, parietal cortex, and dorsal raphe, establishing a coherent corticolimbic pattern of trait-level serotonergic receptor imbalance.

Regarding the dopamine system, Frank et al. (2005) documented elevated D2/D3 receptor binding potential in the anteroventral striatum of recovered AN patients using [^11^C]raclopride, with the elevation positively correlated with harm avoidance in the dorsal caudate and putamen. The increased D2/D3 receptor binding was interpreted as compensatory upregulation in response to tonic dopamine hypofunction in the mesolimbic circuit (Frank et al., 2005). It is important to note that a controlled [^11^C]raclopride PET study on 21 underweight AN patients and 25 healthy controls found no significant differences in striatal D2 receptor binding potential, with no detectable change after weight restoration in the 15 patients completing both scans (Broft et al., 2015). This null finding warrants explicit reconciliation with the elevated binding reported by Frank et al. (2005) as the two studies differ along three methodologically consequential dimensions: illness phase (active *versus* recovered), region-of-interest segmentation (whole striatum *versus* anteroventral focus), and the temporal scale on which compensatory receptor remodeling is detectable. It seems feasible to suggest that the elevated binding reported by Frank et al. (2005) reflects a trait-level compensatory upregulation that emerges progressively across the illness course and is most readily detected in weight-recovered patients, whereas the null finding reported by Broft et al. (2015) reflects either an earlier disease stage at which compensation has not yet developed or a measurement window in which the [^11^C]raclopride signal-to-noise ratio is insufficient to resolve a subregional D3-preferential effect against the D2-dominated whole-striatum binding pool. It is also worthy of notice that [^11^C]raclopride may not be well suited to separating D3-specific effects from the broader D2-dominant striatal signal. Accordingly, the [^11^C]raclopride literature in AN is best characterized as state- and stage-dependent rather than uniformly negative, and the Broft null finding should temper, rather than refute, the trait-level dopaminergic abnormality posited here.

Further corroborating evidence for the dopaminergic trait abnormality in AN comes from a PET study on 27 individuals recovered from eating disorders, in whom a significant positive correlation between serotonin transporter binding ([^11^C]McN5652) and D2/D3 receptor binding ([^11^C]raclopride) was identified in the dorsal caudate, anteroventral striatum, middle caudate, and ventral putamen—a pattern that was entirely absent in healthy controls (Bailer et al., 2013). Linear regression analysis demonstrated that the interaction between the two radioligand measures in the dorsal putamen significantly predicted harm avoidance scores. This corroborates the original findings of Frank et al. (2005) and points to the anteroventral striatum as the primary locus of dopaminergic trait abnormality in AN. Accordingly, these data posit that dopaminergic and serotonergic systems do not operate in isolation in AN; rather, their co-dysregulation, manifested as parallel upregulation of striatal D2/D3 binding and serotonin transporter binding, jointly underlies the elevated harm avoidance that constitutes AN’s most pharmacologically tractable trait vulnerability.

### 2.4 SPECT: regional cerebral blood flow

SPECT studies on AN have consistently identified temporal lobe hypoperfusion as the most reproducible regional cerebral blood flow (rCBF) abnormality. A study of 21 individuals with teenage-onset AN—19 of whom were weight-restored at a follow-up conducted 7 years after onset—found marked hypoperfusion in temporal, parietal, occipital, and orbitofrontal lobes, with rCBF correlating with BMI, suggesting persistent hypoperfusion independent of nutritional state (Råstam et al., 2001). Further corroborating evidence comes from a sample of 15 newly referred children and adolescents, 73% of whom showed asymmetric hypoperfusion in at least one brain area, most frequently the temporal lobe, followed by the parietal lobe suggesting an imbalance of neural circuits within the limbic system (Chowdhury et al., 2003).

Anterior cingulate hypoperfusion at rest in restricting-type AN (Naruo et al., 2001) assumes particular clinical significance as it has been linked to AN’s most treatment-resistant features: the relentless pursuit of thinness and marked body image distortion. A three-condition SPECT study found that AN patients showed hyperactivation of the left parietal and right superior frontal cortex during filmed body image exposure, with left parietal rCBF changes being specifically associated with body distortion severity (Beato-Fernández et al., 2009), implicating the parietal cortex in the storage or active processing of a distorted body schema. In weight-recovered AN patients, SPECT findings become more heterogeneous, with some studies reporting normalization and others—particularly in restricting-type—reporting persistent hypoperfusion.

In summary, four imaging modalities converge on a coherent picture of tonic mesolimbic hypodopaminergia, persistent serotonergic trait abnormalities across both 5-HT1A and 5-HT2A receptor systems, prefrontal overengagement, and cerebral hypoperfusion in temporal and cingulate circuits. This convergent neurobiological signature constitutes the substrate upon which the mechanistic rationale for cariprazine use is discussed.

## 3 Cariprazine: pharmacological profile relevant to anorexia nervosa

Cariprazine (Vraylar, Allergan; Reagila, Gedeon Richter) is an oral atypical antipsychotic, with a receptor profile distinguishing it from all currently available agents. It is a D3-preferring D3/D2 receptor partial agonist—with approximately 10-fold greater binding affinity for the D3 receptor (pKi 10.07) than for the D2 receptor (pKi 9.16–9.31)—a partial agonist at the 5-HT1A receptor (pKi 8.59), and an antagonist at 5-HT2A (pKi 7.73) and 5-HT2B receptors (Citrome, 2016; Huang et al., 2019). This receptor fingerprint is clinically expressed as efficacy against positive symptoms, negative symptoms, anhedonia, and cognitive deficits in schizophrenia and bipolar disorder, thereby affording a pharmacological toolkit directly relevant to the AN phenotype (McIntyre et al., 2025; Citrome, 2016; Koziej et al., 2024).

As a partial agonist, cariprazine functions as a “dopamine stabilizer”: when endogenous dopamine tone is low—as in mesolimbic hypodopaminergia—cariprazine functionally acts as an agonist, enhancing receptor activation; when dopamine levels are high, it acts as a functional antagonist. This intrinsic property renders cariprazine’s effects inherently circuit-specific and self-limiting, properties of utmost significance in AN, where the dopaminergic perturbation is regionally asymmetric.

### 3.1 D3 receptor partial agonism and mesolimbic dopamine

D3 receptors are concentrated in the nucleus accumbens, ventral striatum, and limbic areas that constitute the mesolimbic reward circuit—a system consistently perturbed in AN neuroimaging studies. Preclinical microdialysis studies demonstrated that cariprazine significantly increased dopamine, norepinephrine, and serotonin efflux in the rat nucleus accumbens and hippocampus and, furthermore, increased glutamate and glycine efflux in the nucleus accumbens (Huang et al., 2019). The D3 receptor mediation of these effects was established by an occlusion paradigm: pre-administration of the full D3 agonist 7-OH-DPAT, which maximally engages the D3 receptor pool, abolished cariprazine’s subsequent capacity to further modulate neurotransmitter efflux, confirming that cariprazine and the full agonist act upon a common receptor population and that cariprazine’s action is D3-mediated.

The primary mechanism by which cariprazine is predicted to restore mesolimbic dopamine signaling in AN resides in postsynaptic D3 receptor partial agonism: under the hypodopaminergic conditions prevailing in the AN ventral striatum, cariprazine’s intrinsic efficacy at D3 receptors (Kiss et al., 2010) produces net agonist activation of postsynaptic D3 receptors in the nucleus accumbens. This effect that may translate into restoring incentive salience to food cues—a mechanism corroborated by the D3-receptor-dependent antianhedonic effect established in knockout experiments (Duric et al., 2017). A secondary, substrate-dependent presynaptic contribution is also plausible. Cariprazine partially activates presynaptic D3 autoreceptors, which, under conditions of low endogenous dopamine tone, are submaximally occupied by the endogenous ligand. As cariprazine’s intrinsic efficacy at this site is low, the resulting autoreceptor activation is submaximal relative to what a full agonist would achieve, and it may produce a net increase in terminal dopamine efflux relative to the hypodopaminergic baseline—although this effect is substrate-dependent and secondary to the postsynaptic mechanism. The D3-receptor mediation of the dopamine efflux increase was established by the Huang et al. (2019). They showed that cariprazine’s effect may be abolished by pre-administration of the full D3 agonist 7-OH-DPAT, a finding that confirmed a common D3 receptor population underscoring the net dopaminergic output, although this paradigm could not dissociate presynaptic from postsynaptic contributions. The proposed primary mechanism of cariprazine in AN is postsynaptic D3 partial agonism in the nucleus accumbens (Batinic et al., 2021). A secondary presynaptic autoreceptor contribution is mechanistically plausible but contingent upon the degree of hypodopaminergic substrate state.

Cariprazine’s antianhedonic properties have been demonstrated in a chronic mild stress model of depression. Papp et al. (2014) found that cariprazine significantly reversed chronic mild stress-induced anhedonia—measured by sucrose preference—in rats, and this antianhedonic effect was absent in D3-receptor-knockout mice, establishing D3-receptor-dependent mediation (Duric et al., 2017). Clinically, *post-hoc* analysis of three randomized placebo-controlled trials in bipolar I depression confirmed that cariprazine produces a significant improvement in anhedonia symptoms (McIntyre et al., 2025). It is suggested that this antianhedonic action operates not through food-specific changes but through restoration of mesolimbic D3-mediated incentive salience to naturally rewarding stimuli; food, being among the most salient natural rewards, would thereby regain motivational traction once the underlying D3 receptor signal is normalized, without requiring the drug itself to encode food specificity. This convergence renders the antianhedonic mechanism the most directly supported pharmacological prediction of the present hypothesis.

### 3.2 Prefrontal glutamate modulation

A microdialysis study using a phencyclidine (PCP) rat model of schizophrenia demonstrated that cariprazine dose-dependently attenuated PCP-induced increases in glutamate, dopamine, noradrenaline, and serotonin in the medial prefrontal cortex (mPFC), exhibiting approximately five-fold more potency than aripiprazole (Kehr et al., 2018). This prefrontal glutamate-dampening effect assumes direct relevance to AN as the disorder is characterized by excessive mPFC engagement during reward processing—a pattern that cariprazine’s normalizing, rather than fully suppressing, action would be predicted to attenuate. As cariprazine is a partial agonist operating *via* state-dependent pharmacology, it attenuates hyperactivated signaling while preserving a physiological baseline activity, thereby correcting the excess without producing the opposite extreme of prefrontal inhibition. Furthermore, this mechanism confers a degree of selectivity because the partial agonist effect is modulatory rather than ablative *per se*; for example, its maximum effect is limited by its partial efficacy, allowing it to function as a functional antagonist in hyperactivated circuits while preserving a baseline level of signaling in circuits operating at physiological tone (Ohlsen and Pilowsky, 2005). Hence, it is unlikely to impair the prefrontal functions that are intact in AN.

### 3.3 Serotonergic actions: trait-level influences

Cariprazine’s 5-HT1A partial agonism produces downstream effects on prefrontal dopamine and acetylcholine efflux, contributing to pro-cognitive and anxiolytic effects. Moreover, chronic cariprazine administration was shown to improve learning associated with the hippocampus, the amygdala, and the prefrontal cortex (Zlatanova et al., 2022). Conversely, cariprazine’s 5-HT2A antagonism (Selvan et al., 2024) may additionally reduce the anxiety- and harm-avoidance-mediating effects of residual 5-HT2A receptor signaling. However, given that 5-HT2A binding is already significantly reduced in recovered AN as a trait marker, this action warrants careful consideration: it may provide further modulation of an already attenuated signal, which could be either therapeutically beneficial (reducing harm avoidance) or neutral.

Nonetheless, the directional consequence of cariprazine’s 5-HT1A engagement requires explicit articulation as state- and region-dependent rather than uniformly inhibitory. As a partial agonist with low intrinsic efficacy at 5-HT1A receptors (pK_i_ 8.59: Kiss et al., 2010), cariprazine functions as a 5-HT1A stabilizer analogous to its dopamine stabilizer property at D3/D2 receptors: where endogenous 5-HT tone is supraphysiological—as in the trait-amplified corticolimbic substrate of AN, in which 5-HT1A binding is elevated by 30%–70% across cingulate, frontal, parietal, temporal, and dorsal raphe regions (Bailer et al., 2007)—the partial agonist competes with the full-agonist endogenous ligand at postsynaptic receptors and imposes a pharmacological ceiling, thereby acting as a functional antagonist that dampens the trait-amplified inhibitory tone. Conversely, under the circumstances of normal or attenuated 5-HT tone, the same compound exerts net agonist activity, as documented by the full-agonist action at postsynaptic hippocampal 5-HT1A receptors (Herman et al., 2018), and the increased tonic 5-HT1A neurotransmission following chronic administration (El Mansari et al., 2020), the latter detailed in Section 4.3. These findings suggest that this bidirectional 5-HT1A action is mediated through two complementary mechanisms. First, at postsynaptic corticolimbic 5-HT1A receptors operating under supraphysiological endogenous tone, competitive occupancy by the partial agonist curtails the dominant inhibitory-hyperpolarizing gating that produces the harm-avoidant phenotype (Araneda and Andrade, 1991). Second, at presynaptic somatodendritic 5-HT1A autoreceptors on dorsal raphe neurons—themselves trait-amplified in AN (Bailer et al., 2007)—chronic partial-agonist occupancy progressively desensitizes the autoreceptor brake on ascending serotonergic firing, thereby redistributing net 5-HT delivery to forebrain targets in a manner mechanistically analogous to chronic selective serotonin reuptake inhibitor (SSRI) action. Accordingly, the apparent paradox that cariprazine simultaneously dampens cortical 5-HT1A signaling and increases hippocampal 5-HT1A neurotransmission is resolved by recognizing that these effects occur at distinct receptor populations under distinct tone conditions, jointly converging upon a network-level recalibration toward a normalized inhibitory–excitatory balance.

On this account, a hypothesis may be formulated that cariprazine would recapitulate the net anxiolytic effect of tryptophan-limiting starvation by a mechanistically distinct route. The starvation-induced anxiolysis described by Kaye et al. (2003) is achieved by lowering endogenous 5-HT availability and thereby reducing total ligand drive at the trait-amplified 5-HT1A receptor pool; cariprazine, conversely, is predicted to leave endogenous ligand availability unchanged while occupying the trait-amplified postsynaptic 5-HT1A pool with a low-intrinsic-efficacy partial agonist that competes with full-agonist endogenous 5-HT and consequently caps the inhibitory cortical signal at a sub-supraphysiological ceiling. Furthermore, the parallel desensitization of presynaptic somatodendritic 5-HT1A autoreceptors over the chronic-administration window (El Mansari et al., 2020) progressively redistributes net 5-HT delivery to forebrain targets, in a manner mechanistically analogous to chronic SSRI action but acting upon a receptor population whose trait-level density is itself the source of dysregulation. Nonetheless, the net direction of cariprazine’s effect upon corticolimbic excitability in the AN substrate cannot be inferred with certainty from the available rodent electrophysiology since the supraphysiological-tone substrate that the AN brain is hypothesized to instantiate has not been directly modeled in the chronic-dosing studies. This prediction, therefore, constitutes the central serotonergic therapeutic rationale for cariprazine in AN while remaining contingent upon prospective clinical validation and is further corroborated by the additional receptor-profile features (5-HT2A antagonism and D3-preferring partial agonism) that address multiple dimensions of the disorder.

### 3.4 5-HT2A antagonism: modest adjunctive effect

Cariprazine’s 5-HT2A antagonism (Kiss et al., 2010) may additionally reduce the harm-avoidance signaling mediated through residual 5-HT2A receptor activity. However, given that 5-HT2A binding is already significantly reduced in recovered AN as a trait marker (Frank et al., 2002; Bailer et al., 2004), this action warrants careful consideration: the drug would superimpose functional antagonism upon an already attenuated receptor population. Any residual harm-avoidance signaling mediated through 5-HT2A may be modestly attenuated by this superimposition, although the magnitude of clinical benefit remains uncertain. Accordingly, the 5-HT2A action is best characterized as a probable adjunct to, rather than the principal mechanism of, the predicted clinical response.

### 3.5 Substrate-dependent D3 set-point recalibration

Chronic cariprazine administration uniquely confers a substrate-dependent remodeling of mesolimbic D3 receptor density that plays a central role in consolidating its antianhedonic mechanism. In the unperturbed rodent substrate, 14-day administration produced dose-dependent increases in D3 receptor levels in the nucleus accumbens shell (31%–48%) and islands of Calleja (32%–57%), paralleling increases in D2 receptor levels in the prefrontal cortex and striatum (Choi et al., 2014). This action has been proposed as a common neurobiological mechanism of antidepressant treatment more broadly, in which D3 receptor expression in the nucleus accumbens is increased as a final-common downstream consolidation step (Lammers et al., 2000). However, the directional outcome of this remodeling is not pharmacologically pre-determined but rather governed by the substrate state, in a manner mechanistically analogous to the dopamine stabilizer property elaborated in Section 5. In the healthy substrate, where baseline D3 tone is normal, sustained low-level partial-agonist occupancy drives receptor density toward an intermediate set point that lies above the baseline and manifests as the upregulation documented by Choi et al. (2014). Conversely, in AN substrate—in which mesolimbic D2/D3 binding is already compensatorily upregulated in response to tonic dopaminergic hypofunction (Frank et al., 2005)—chronic partial-agonist occupancy supplies a receptor-stimulation signal that the hypofunctional endogenous ligand has failed to deliver. It may be postulated, on the basis of homeostatic logic and by analogy to the directional reversibility of receptor remodeling documented for chronic SSRI exposure, that this exogenous signal would relieve the drive sustaining the compensatory upregulation, with the predicted consequence of receptor-density normalization rather than further upregulation. This prediction has no direct empirical support in AN; the data reported by Choi et al. (2014) demonstrate upregulation in the unperturbed rodent striatum and cannot be extrapolated with certainty to a pre-upregulated substrate. Accordingly, the substrate-dependent reversal posited here is best framed as a falsifiable prediction for prospective PET studies in cariprazine-treated AN—in which the predicted signature is a reduction in [^11^C]raclopride binding potential in the anteroventral striatum following chronic exposure. In summary, cariprazine’s chronic D3 effect is plausibly characterized as a set-point recalibration mechanism whose directional outcome is hypothesized to depend on the substrate state, with the consolidation of antianhedonic action expected to be preserved across substrate conditions; the directional component, however, awaits direct neuroimaging tests.

### 3.6 Safety considerations in AN: a comparative advantage

Cariprazine’s safety profile affords a significant comparative advantage over existing pharmacotherapy for AN. The risk of weight gain is markedly lower than that associated with olanzapine: in schizophrenia trials, only approximately 8% of patients receiving cariprazine at doses of 1.5–6 mg/day gained more than 7% of baseline body weight, compared with 5% of patients receiving placebo, yielding a number needed to harm (NNH) of 34 (Citrome, 2018). This compares very favorably with olanzapine, where the NNH for clinically significant weight gain (≥7%) is estimated to 6. No clinically meaningful effects on prolactin, QTc interval, or metabolic parameters have been identified with cariprazine (Citrome, 2018), further underscoring its suitability in a population already at risk for metabolic and cardiac complications secondary to malnutrition.

The principal safety concern is akathisia, which is common with cariprazine (NNH approximately 10–19 depending on dose), a potentially distressing symptom in a population characterized by high anxiety and physical hyperactivity. Accordingly, dose optimization favoring the lower therapeutic range of 1.5–3 mg/day is recommended, and a protocol-defined management algorithm should be implemented [e.g. implementing a rating scale with established clinical utility, validity, and reliability such as the Barnes Akathisia Rating Scale (BARS), the most widely used rating scale designed to identify and assess antipsychotic-induced akathisia (Pringsheim et al., 2018). A global score ≥2 would trigger dose reduction from 3 mg/day to 1.5 mg/day, and a BARS global score ≥3 would trigger consideration of drug cessation and initiation of propranolol at 10–20 mg/day (Barnes, 1989; 2003)]. Extrapyramidal effects are otherwise low, attributable to cariprazine’s preferential ventral striatal D3 localization.

## 4 Mechanistic hypothesis: predicted neuroimaging effects of cariprazine in AN

Based on the receptor-pharmacological profile articulated in Section 3 and the multimodal neuroimaging signature documented in Section 2, the present section advances testable mechanistic predictions for the neuroimaging consequences of cariprazine administration in AN. The predictions are organized along the same four modality axes (fMRI, EEG, PET, and SPECT), so that each imaging-defined abnormality maps onto a corresponding cariprazine-mediated correction. D3 partial agonism is predicted to restore ventral striatal reward signaling on fMRI; the combined actions of D3 and 5-HT1A, with their downstream cholinergic consequences, are predicted to normalize the P300/N2 inhibitory-control deficit and the parieto-occipital theta hyperarousal signature on EEG; chronic D3-mediated set-point recalibration, together with 5-HT1A network modulation, is predicted to recalibrate the trait-level receptor-binding abnormalities documented on PET, and pro-dopaminergic disinhibition in mesolimbic and anterior cingulate circuits is predicted to attenuate the SPECT-documented hypoperfusion. It seems feasible to suggest that this modality-by-modality mapping provides a directly testable framework for prospective clinical neuroimaging trials of cariprazine in AN. The predicted effects of cariprazine on neuroimaging abnormalities in AN are summarized in Table 2 and Figure 1.

**TABLE 2:** Predicted effects of cariprazine on neuroimaging abnormalities in anorexia nervosa.

Modality	Neuroimaging abnormality in AN	Predicted cariprazine effect	Pharmacological basis
fMRI	Blunted ventral striatal and amygdala activation in response to food-related stimuli.	Increased BOLD prediction-error signal in the nucleus accumbens and amygdala during food exposure; restoration of incentive salience to food cues. The corresponding reduction in tonic-versus-phasic contrast for non-food cues is predicted to attenuate the BOLD response to thinness-related visual feedback and exercise cues that sustains the addiction-like reinforcement of restriction.	D3 partial agonism in the nucleus accumbens restores dopaminergic tone primarily through postsynaptic D3 receptor net agonism under hypodopaminergic conditions; the partial-agonist intrinsic efficacy ceiling precludes the overstimulation of normally functioning circuits.
fMRI	Excessive prefrontal and dorsal striatal overactivation during reward and habit tasks, reflecting exaggerated cognitive control over eating.	Normalization of overactivation; attenuation of top-down inhibition of food reward without inducing impulsivity.	Prefrontal glutamate-dampening action of cariprazine; partial-agonist character functions as a functional antagonist in hyperactivated circuits while preserving baseline activity in circuits operating at physiological tone.
EEG	Attenuated P300 amplitudes and delayed N2 latencies, indexing fronto-central dysfunction in action monitoring and inhibitory control.	Improved efficiency of prefrontal information processing indexed by these ERP components, in line with cariprazine’s documented pro-cognitive effects in schizophrenia.	D3 partial agonism in prefrontal circuits combined with 5-HT1A-mediated increases in cortical acetylcholine efflux.
EEG	Resting-state cortical hyperarousal: elevated parieto-occipital theta amplitude with reduced-alpha/increased-beta signature.	Normalizing effect on the theta hyperarousal profile, without eliminating alpha-rhythm contributions to attention; testable using resting-state EEG within a clinical trial.	5-HT1A partial agonism at raphe autoreceptors modulates downstream serotonergic output, broadly reducing cortical excitability.
PET	Reduced 5-HT2A binding as a trait marker in recovered AN.	Modest attenuation of residual 5-HT2A-mediated harm-avoidance signaling; magnitude of clinical benefit uncertain given the trait-level reduction in receptor density.	Functional 5-HT2A antagonism superimposed upon an already attenuated receptor population.
PET	Trait-elevated 5-HT1A binding across cingulate, frontal, parietal, temporal, and dorsal raphe regions.	Bidirectional 5-HT1A action: dampening of supraphysiological corticolimbic inhibitory tone responsible for harm avoidance; full-agonist action at postsynaptic hippocampal 5-HT1A receptors and increased tonic 5-HT1A neurotransmission in the hippocampus following chronic administration; progressive desensitization of presynaptic somatodendritic 5-HT1A autoreceptors on dorsal raphe neurons.	5-HT1A stabilizer property of a partial agonist with low intrinsic efficacy (pKi 8.59): it functions as a functional antagonist where endogenous 5-HT tone is supraphysiological and as a net agonist where tone is normal or attenuated.
PET	Elevated D2/D3 receptor binding in the anteroventral striatum (compensatory upregulation in response to tonic dopaminergic hypofunction).	Substrate-dependent set-point recalibration of receptor density toward an intermediate level below the disease state—manifesting as receptor-density normalization rather than further upregulation.	Chronic D3 partial agonism supplies the receptor-stimulation signal that the endogenous ligand fails to deliver, relieving the homeostatic drive that sustains the compensatory upregulation; partial-agonist intrinsic efficacy precludes runaway hyperdopaminergic stimulation.
SPECT	Temporal and anterior cingulate hypoperfusion.	Increased metabolic activity and regional cerebral blood flow in limbic and temporal structures; partial restoration of metabolic activity in the anterior cingulate.	Pro-dopaminergic action via D3 partial agonism increases dopamine efflux in the nucleus accumbens and hippocampus; being a partial agonist, cariprazine competes with submaximal endogenous dopamine at inhibitory autoreceptors in the VTA and dampens dopamine release inhibition in anterior cingulate-projecting circuits.
SPECT	Parietal hypoperfusion linked to body image distortion.	Indirect normalization through a reduction in incentive salience attributed to distorted body representations processed in parietal cortex; no direct pharmacological action on the parietal body schema is proposed.	Downstream consequence of mesolimbic and cingulate circuitry normalization rather than direct receptor-level engagement of the parietal cortex.

### 4.1 fMRI: restoring striatal reward signaling

Cariprazine’s D3 partial agonism is predicted to restore dopaminergic tone in the nucleus accumbens: under the low-tone conditions prevailing in the AN ventral striatum, cariprazine’s low intrinsic efficacy translates into net agonist activity at postsynaptic D3 receptors, which is proposed to restore incentive salience to food cues. This mechanism is corroborated by the preclinical demonstration that the antianhedonic effect of cariprazine is D3-receptor-dependent (Duric et al., 2017; Huang et al., 2019). A secondary presynaptic contribution is mechanistically plausible but subsidiary and substrate-dependent.

This mechanism predicts an increased BOLD prediction-error signal in the nucleus accumbens and amygdala during food exposure—by elevating the tonic dopaminergic floor against which food-cue phasic responses are evaluated—thereby restoring a degree of incentive salience to food. Critically, the corresponding reduction in tonic-versus-phasic contrast for non-food cues is predicted to attenuate the BOLD response to thinness-related visual feedback and exercise cues that sustains the addiction-like reinforcement of restriction (Verhagen et al., 2009; Foldi et al., 2017). Critically, the same mechanism would not be expected to overstimulate dopaminergic circuits that function normally as the partial agonist characteristic of cariprazine confers a pharmacological ceiling effect—a feature of particular relevance in AN, where dopaminergic excess in non-ventral striatal circuits could worsen anxiety or drive compulsive behaviors (Mota et al., 2025).

The excessive frontal and dorsal striatal overactivation observed in AN fMRI— reflecting exaggerated cognitive control over eating—is predicted to normalize through cariprazine’s prefrontal glutamate-dampening action (Kehr et al., 2018). The moderating, rather than abolishing, characteristic of this effect is consistent with the partial agonist mechanism and would attenuate top-down inhibition of food reward without inducing impulsivity.

### 4.2 EEG: improving inhibitory control and normalizing cortical arousal

The P300 amplitude deficits and delayed N2 latencies in AN reflect fronto-central hypofunction in action monitoring and inhibitory control—processes that partially depend on dopaminergic and cholinergic inputs to the prefrontal cortex (Amalric et al., 2021). Cariprazine’s D3 partial agonism in prefrontal circuits, combined with its 5-HT1A-mediated increases in cortical acetylcholine efflux, would be expected to improve the efficiency of prefrontal information processing indexed by these ERP components. This is in line with cariprazine’s documented pro-cognitive effects in schizophrenia, where similar fronto-central deficits are amenable to pharmacological correction. The parieto-occipital theta hyperarousal signature that persists after refeeding (Hatch et al., 2011; Spalatro et al., 2021) reflects, in part, the trait-amplified high-gain inhibitory serotonergic system; the 5-HT1A stabilizer action of cariprazine at trait-amplified postsynaptic sites and progressive desensitization of presynaptic autoreceptors are predicted to attenuate this hyperarousal profile, without eliminating alpha-rhythm contributions to attention. This hyperarousal signature may, at least in part, reflect serotonergically driven cortical overexcitability—a hypothesis that remains to be evaluated directly. Cariprazine’s 5-HT1A partial agonism at raphe autoreceptors modulates downstream serotonergic output, and 5-HT1A agonism broadly reduces cortical excitability in preclinical models (Bailer et al., 2007). Accordingly, a normalizing effect on the theta hyperarousal profile—without eliminating the alpha rhythm contributions to attention—represents a mechanistically plausible EEG prediction, and a prediction that may be directly tested using resting-state EEG within a clinical trial.

### 4.3 PET: serotonergic and dopaminergic receptor normalization

The reduced 5-HT2A binding documented as a trait marker in recovered AN is not a direct pharmacological target for cariprazine’s 5-HT2A antagonism in the conventional sense; rather, cariprazine would superimpose functional antagonism upon an already attenuated receptor population. Any residual harm-avoidance signaling mediated through 5-HT2A may be modestly attenuated by this superimposition, although the magnitude of clinical benefit is uncertain given the trait-level reduction in receptor density. Conversely, the principal serotonergic mechanism by which cariprazine is predicted to confer benefit in AN resides in its partial agonism at 5-HT1A receptors. Corroborating evidence shows functionally a full-agonist action at postsynaptic hippocampal 5-HT1A receptors after both acute and chronic administration (Herman et al., 2018; El Mansari et al., 2020). Furthermore, chronic (14-day) cariprazine has been shown to increase tonic 5-HT1A receptor-mediated neurotransmission in the hippocampus (El Mansari et al., 2020), which may underlie the anxiolytic and cognitive components of the predicted clinical response.

Regarding the dopaminergic PET findings, the elevated D2/D3 binding in the anteroventral striatum in recovered AN—interpreted as compensatory receptor upregulation in response to tonic hypodopaminergia—represents a directly accessible pharmacological target. Consistent with the substrate-dependent set-point logic articulated in Section 3.4, the elevated D2/D3 binding in AN—which represents pre-existing compensatory upregulation rather than a state of receptor preservation—is the very feature that renders the dopaminergic trait abnormality pharmacologically tractable to chronic D3 partial agonism.

### 4.4 SPECT: increasing regional cerebral blood flow in hypoperfused circuits

SPECT consistently identifies temporal and cingulate hypoperfusion in AN. The pro-dopaminergic action of cariprazine in the mesolimbic circuit—increasing dopamine efflux in the nucleus accumbens and hippocampus—would be expected to increase metabolic activity and regional cerebral blood flow in limbic and temporal structures. The anterior cingulate, a region of consistently reduced rCBF in restricting-type AN, receives dopaminergic projections from the ventral tegmental area (VTA). Cariprazine’s D3 partial agonism—acting as a net agonist at postsynaptic D3 receptors and, under low-tone conditions, partially activating presynaptic D3 autoreceptors in the VTA—is predicted to increase dopaminergic tone in anterior cingulate-projecting circuits and partially restore metabolic activity in this hypoperfused structure. For parietal hypoperfusion linked to body image distortion, the mechanistic prediction is more indirect: the normalization of mesolimbic and cingulate circuitry may reduce the incentive salience attributed to distorted body representations processed in parietal cortex, although a direct pharmacological action on the parietal body schema *per se* is not proposed.

## 5 Discussion

The mechanistic hypothesis articulated in the preceding sections invites broader contextualization in light of the existing pharmacological landscape, the conceptual neuroscience of reward and incentive salience, the temporal architecture of AN’s neurobiology, and the limitations inherent in extrapolating from receptor-level pharmacology to the clinical phenotype. Accordingly, the present discussion is organized around five complementary considerations: cariprazine’s mechanistic advantages relative to agents currently used off-label in AN; the relationship between food reward hyposensitivity and the broader constructs of anhedonia and incentive salience; the trait-versus-state distinction that bears upon optimal treatment timing; the regional and temporal asymmetries afforded by cariprazine’s dopamine stabilizer property; and the limitations and uncertainties that must constrain interpretation. Each of these considerations underscores a distinct facet of why cariprazine’s receptor pharmacology may be uniquely suited to AN while also delineating the boundaries within which the present hypothesis remains exploratory and dependent on prospective clinical validation.

### 5.1 Mechanistic advantages relative to currently used agents

The pharmacological rationale for cariprazine in AN is distinct from, and mechanistically superior to, the rationale for agents currently used off-label. Olanzapine—the best-studied agent in AN—acts primarily as a D2 antagonist and has H1, M1, and alpha-1 blocking properties that produce weight gain and sedation. In the largest randomized controlled trial so far, olanzapine produced only a 0.6 kg/m^2^ greater increase in BMI than placebo after 16 weeks, with no significant benefit in eating disorder psychopathology (Attia et al., 2019). In AN, where dopaminergic hypofunction in the reward circuit underlies the core neurobiology, blocking D2 receptors further reduces dopaminergic tone in the mesolimbic system—the converse of what the neuroimaging evidence posits is required. Accordingly, olanzapine’s clinical effects in AN may be mediated primarily through its weight-promoting and anxiolytic (H1 blockade) properties rather than through neurobiologically targeted action.

SSRIs and SNRIs address serotonergic dysfunction but with mechanisms that are poorly matched to AN’s specific receptor-level alterations. Antidepressant trials have consistently failed to demonstrate efficacy in underweight AN patients, and the hyperserotonergic state, along with downregulation of 5-HT2A binding in AN, suggests that augmenting serotonergic tone may have limited efficacy at a receptor that is already underexpressed. Furthermore, the absence of direct dopaminergic action means that SSRIs do not address the reward circuit hypofunction that assumes the role of AN’s most consistent neurobiological signature.

Furthermore, an apparent asymmetry between the serotonergic and dopaminergic limbs of the present model warrants explicit reconciliation, as does the cue-selective character of the dopaminergic abnormality itself. The serotonergic limb plays a central role in casting restriction as ego-syntonic by lowering an aversive premorbid 5-HT tone, whereas the dopaminergic limb posits that restriction occurs against a background of tonic mesolimbic hypodopaminergia that renders food motivationally inert. These two mechanisms are not mutually exclusive but operate on different cue sets and at different stages: the serotonergic anxiolytic relief is recruited at the moment of food avoidance, whereas the dopaminergic mechanism accounts the failure of food cues to motivate intake in a cue-selective manner. Cue-selective, rather than uniformly suppressed, mesolimbic dopaminergic responsiveness in AN is supported by some evidence at both preclinical and clinical levels. The most direct human neuroimaging support derives from Fladung et al. (2013), who demonstrated exaggerated ventral striatal BOLD activation in response to underweight body-image stimuli in early-illness adolescent AN—establishing, within a single fMRI paradigm, that the same mesolimbic circuit that shows blunted activation to food stimuli (Bronleigh et al., 2022) retains or amplifies its response to thinness-related visual cues. At the preclinical level, Verhagen et al. (2009) demonstrated that dopamine release from the nucleus accumbens was not elevated during the initiation of food-anticipatory behavior in the activity-based anorexia rodent model despite marked starvation and hyperactivity—corroborating the failure of food cues to recruit mesolimbic dopamine—whereas Foldi et al. (2017) showed that chemogenetic activation of the VTA-to-nucleus accumbens projection rescued body weight by selectively augmenting food intake, establishing a causal role for mesolimbic hypodopaminergia in the food-motivational deficit. It may be postulated, therefore, that the addiction-like, perfectionistic pursuit of thinness in AN is not paradoxical for a system with selectively low food-cue dopaminergic responsiveness since the goal-directed behavior is reinforced by an entirely separate and operationally intact mesolimbic dopaminergic response to thinness cues and exercise (Ziemichód et al., 2026). It must be acknowledged, however, that the specific inference that exercise cues recruit a preserved or augmented dopaminergic response in human AN has not been directly established by PET or fMRI: the human evidence reported by Fladung et al. pertains to body-image stimuli in adolescent early-illness patients, and generalization to exercise-related cue processing and to the chronic trait-level dopaminergic state is inferential. It is, therefore, proposed that the cue-selective hypodopaminergia formulation represents a mechanistically coherent and preclinically supported hypothesis that organizes the available evidence, but it must be acknowledges that definitive human neuroimaging evidence—specifically, a within-subject PET or fMRI paradigm comparing ventral striatal responses to food *versus* exercise cues in the same AN sample—remains to be obtained.

The cue-selectivity formulation, however, raises a question that the dopamine-enhancement prediction must address: if non-food-cue dopaminergic responsiveness is already preserved or augmented in active AN, would cariprazine’s D3 partial agonism not attenuate, rather than augment, that signaling? This question may be resolved by recognizing that the cue-selective abnormality in AN resides not in differential receptor populations but in the contrast between phasic cue-evoked dopamine release and the tonic background dopaminergic floor against which it is evaluated. In active AN, tonic mesolimbic dopamine is reduced—corroborated by the compensatory upregulation of D2/D3 binding documented by PET (Frank et al., 2005)—while phasic responses to thinness and exercise cues remain operationally intact and accordingly stand out against this depressed tonic floor with disproportionate motivational salience. Phasic responses to food cues, conversely, are themselves attenuated against the same depressed floor and consequently fail to recruit incentive salience. Cariprazine’s chronic D3 partial agonism is predicted to increase tonic dopamine efflux under hypodopaminergic baseline conditions (Huang et al., 2019), while its low intrinsic efficacy precludes the recruitment of the maximal receptor activation that phasic cue-evoked release achieves. The net effect, accordingly, is bidirectional in relative, but not absolute, terms: food-cue phasic responses, restored against the elevated tonic floor, regain a degree of motivational traction; non-food-cue phasic responses, evaluated against the same elevated floor, lose a portion of the disproportionate contrast that previously rendered them dominant. Furthermore, the partial agonist ceiling property ensures that no cue domain is pharmacologically abolished since the maximal receptor stimulation cariprazine can recruit is bounded by its intrinsic efficacy. This is corroborated by the broader pharmacological principle that partial agonists narrow the dynamic range of receptor activation around an intermediate set point rather than inverting it (Ohlsen and Pilowsky, 2005). We propose, therefore, that the dopamine stabilizer property of cariprazine operates not only across regional asymmetries between ventral and dorsal striatum but also across temporal asymmetries between tonic background and phasic cue-evoked release within the ventral striatum itself. This dual-axis stabilizer logic renders the cariprazine rationale coherent across both cue domains: tonic floor elevation restores food incentive salience, while the corresponding reduction in tonic-versus-phasic contrast attenuates—without abolishing—the relative motivational dominance of non-food cues that sustains the addiction-like reinforcement of restriction.

Cariprazine is unique in providing simultaneous, mechanistically targeted engagement of both the dopaminergic reward deficit (*via* D3 partial agonism) and the serotonergic trait vulnerability (*via* 5-HT1A partial agonism and progressive receptor upregulation), while its “stabilizer” property confers circuit-level selectivity that prevents the iatrogenic hyperdopaminergia that full agonists would cause. This is underscored by its NNH for clinically significant weight gain of 34, *versus* 6 for olanzapine (for clinically significant weight gain ≥7% from the baseline) (Citrome, 2018)—a comparative advantage of particular relevance in a population for whom weight-gain liability compounds therapeutic anxiety and consequent therapeutic failure.

### 5.2 The dopamine stabilizer property and circuit specificity

Cariprazine’s intrinsic activity as a partial agonist confers mechanistically elegant circuit specificity in AN. In the ventral striatum—where tonic dopaminergic hypofunction prevails—cariprazine acts predominantly as a net agonist, boosting reward signaling. In the dorsal striatum and prefrontal cortex—where dopaminergic tone may be relatively normal or elevated during food-related cognitive control tasks—cariprazine’s partial agonist characteristics suggest that it cannot recruit maximal receptor activation, functioning instead as a modulator that dampens overactivation. This dual capacity—enhancing where there is deficit and moderating where there is excess—is uniquely suited to the regionally asymmetric dopaminergic perturbation identified by fMRI and PET in AN.

No other currently available pharmacological agent possesses this combination of properties. Aripiprazole, the closest structural analog, has approximately 10-fold lower affinity for D3 relative to D2 receptors compared to cariprazine, rendering its actions less concentrated in the nucleus accumbens and limbic reward circuits that are most abnormal in AN. Accordingly, the D3 selectivity of cariprazine is not merely a pharmacological nuisance but a mechanistically decisive property. Furthermore, as elaborated in Section 5.1, the stabilizer property operates not only across regional asymmetries but also across temporal asymmetries between tonic dopaminergic background and phasic cue-evoked release, affording cue-selective recalibration of motivational salience without absolute pharmacological abolition of any cue domain.

### 5.3 The reward deficit hypothesis in AN: relationship between anhedonia and incentive salience

A critical conceptual bridge in this hypothesis is the relationship between the food reward hyposensitivity documented by fMRI and the clinical constructs of anhedonia and incentive salience. Although AN is not classically conceptualized as an anhedonic disorder, the neurobiological evidence increasingly posits that food-specific reward processing is disrupted in AN in a manner neurobiologically analogous to anhedonia in depression, and a meta-analytic synthesis confirms that anhedonia is elevated in AN compared with healthy controls (Dolan et al., 2022). In line with the incentive salience framework proposed by Berridge and Robinson (2016), the dissociation of “wanting” from “liking” affords a parsimonious account of why patients with AN may neither subjectively crave nor hedonically enjoy food while, nonetheless, failing to display generalized anhedonia.

Cariprazine has demonstrated robust antianhedonic efficacy in both preclinical models (Papp et al., 2014; Duric et al., 2017) and in clinical trials in bipolar I depression. The D3-dependent nature of this antianhedonic mechanism—confirmed through knockout experiments (Duric et al., 2017)—directly implicates the nucleus accumbens D3 receptor population that is upregulated in AN. It may be postulated that cariprazine’s antianhedonic action operates not through food-specific changes but through the restoration of mesolimbic D3-mediated incentive salience to naturally rewarding stimuli, with food being among the most salient natural rewards. This would thereby regain motivational traction once the underlying D3-receptor signal is normalized, without requiring the drug itself to encode food-specificity. This convergence renders the antianhedonic mechanism the most directly supported pharmacological prediction of this hypothesis.

### 5.4 Trait versus state: implications for treatment timing

A central tension in the neuroimaging literature concerns whether AN’s brain abnormalities are state-dependent (consequences of malnutrition) or trait-level vulnerabilities. The evidence posits both: structural changes and most fMRI findings reverse with weight restoration, while PET-identified serotonergic receptor abnormalities and some SPECT findings persist in recovered patients.

If cariprazine is administered to severely underweight patients, its action on the reward circuit operates within a brain further compromised by the metabolic and hormonal effects of starvation. Medical stabilization and weight restoration would accordingly be expected to enhance the neurobiological substrate on which cariprazine acts. The optimal treatment window may, therefore, be during the weight restoration and early maintenance phase, when structural brain changes are reversing but trait-level neurobiological vulnerabilities—the serotonergic and dopaminergic receptor abnormalities detected by PET—remain present and pharmacologically accessible. This is in line with the known failure of SSRIs in the acutely underweight phase and their partial efficacy after weight restoration (Crow, 2019). Concomitant CBT-E and nutritional rehabilitation could further optimize this neurobiological substrate (Fairburn et al., 2013).

### 5.5 Limitations and uncertainties

Several important limitations must be acknowledged. First, all neuroimaging predictions in this study are mechanistic extrapolations from receptor pharmacology and preclinical data: no clinical neuroimaging study on cariprazine in AN has been conducted. Second, AN is a heterogeneous disorder; patients with the restricting subtype, those with the binge-purge subtype, and those in different stages of illness may show meaningfully different neurobiological profiles, and the applicability of these predictions across subtypes remains uncertain. The present hypothesis is most directly applicable to the restricting subtype of AN (AN-R) as its principal neuroimaging pillars—elevated D2/D3 binding potential (Frank et al., 2005), trait-level 5-HT1A upregulation (Bailer et al., 2007), and blunted ventral striatal food-cue BOLD signal (Bronleigh et al., 2022)—derive predominantly from restricting-type or undifferentiated samples, and the applicability of these predictions to the binge-purge subtype (AN-BP) requires explicit qualification. The serotonergic limb of the model extends across subtypes: Bailer et al. (2013) demonstrated, in 27 patients with recovered eating disorder including 7 with bulimia-type AN, that the positive correlation between the serotonin transporter and D2/D3 binding potential in the anteroventral striatum—linked to harm avoidance—was present across all eating disorder subgroups and absent in healthy controls. The dopaminergic limb, however, requires further subtype-specific interpretation. In the only fMRI study to directly compare AN subtypes (Murao et al., 2017), 11 AN-R and 12 AN-BP patients were asked to perform a monetary incentive delay task. The authors documented a subtype-divergent dopaminergic neurobiological architecture, reporting heightened punishment-circuit sensitivity rather than a food-reward deficit as the dominant mesolimbic perturbation in AN-BP. This finding is aligned with the results of Bronleigh et al. (2022), whose ALE meta-analysis found that although AN is characterized by decreased amygdala and striatal activation to food stimuli, in bulimia nervosa, striatal and insular food-cue reactivity changes in the opposite direction. In summary, it is proposed that the core dopaminergic mechanism in AN-R does not straightforwardly generalize to AN-BP, requiring additional elaboration.

Third, the weight-gaining properties of cariprazine, while markedly lower than olanzapine (NNH 34 *versus* 6), must be monitored carefully and may be paradoxically advantageous (promoting weight restoration) or, in weight-restored patients, potentially disruptive to relapse prevention. Fourth, the anxiety burden of akathisia in a population characterized by high baseline anxiety, hyperactivity, and somatic vigilance is a genuine safety concern that must be pre-specified and monitored in any trial design. Dosing strategies beginning at 1.5 mg/day with slow titration are accordingly recommended, along with systematic akathisia surveillance using the Barnes Akathisia Rating Scale (Barnes, 1989; 2003) at every visit. Furthermore, as AN commonly begins in adolescence, any future clinical translation of cariprazine under this condition would mandate considering the limited safety data in younger populations. Fifth, AN disproportionately affects adolescents and young adults; cariprazine has limited safety data in pediatric populations. Sixth, the serotonergic predictions (progressive 5-HT1A upregulation) are based on chronic rodent data (Zlatanova et al., 2022) and may not translate linearly to humans in either timescale or magnitude. Nonetheless, even *in lieu* of the limitations, the predictions advanced here are mechanistically grounded but require prospective clinical validation.

## 6 Conclusion

This article presents a mechanistic hypothesis for the repurposing of cariprazine in anorexia nervosa, grounded in a systematic synthesis of multimodal neuroimaging evidence and verified pharmacological data. The convergence between cariprazine’s receptor pharmacology and the neurobiological targets identified by fMRI, PET, SPECT, and EEG in AN is systematic: D3 partial agonism addresses the ventral striatal reward hyposensitivity that underlies the disorder’s fundamental motivational deficit. This affords substrate-dependent set-point recalibration of the trait-elevated D2/D3 receptor population. 5-HT1A partial agonism offers progressive recalibration of the serotonergic trait vulnerability; prefrontal glutamate dampening would normalize excessive cognitive control over eating, and anxiolytic properties may reduce the harm avoidance and anxiety that perpetuate food restriction.

The favorable metabolic profile of cariprazine—NNH for significant weight gain of 34, *versus* 6 for olanzapine—and its mechanistic specificity relative to SSRIs render it a compelling candidate for prospective investigation. No pharmacological agent currently assumes the role of addressing the core neurobiological substrate of AN in the manner mechanistically predicted here. Accordingly, this hypothesis posits a translational pathway of direct scientific and clinical significance, which may pave the way to the first approved pharmacotherapy for this disorder.

## Data Availability

The original contributions presented in the study are included in the article/supplementary material; further inquiries can be directed to the corresponding author.
